# Degenerating Hæmatoma in an Iguana

**Published:** 1882-10

**Authors:** Wm. A. Conklin


REPORTED FROM THE CENTRAL PARK ZOOLOGICAL GARDEN, BY WM. A CONKLIN, D.V.S.

### VI.—DEGENERATING HÆMATOMA IN AN IGUANA.

REPORTED FROM THE CENTRAL PARK ZOOLOGICAL GARDEN, BY WM. A. CONKLIN. D.V.S.

This Iguana had a large swelling on the side for some two months before death. Shortly after the swelling appeared I lanced it, but very little blood or matter flowed from the incision. Up to this time, and after, the animal ate quite well until two days before death. During this time the animal appeared quite well and healthy.

This animal was kept in a cage with some African pythons, and it occurred to me that these snakes in crawling around the cage may have given it a squeeze and thus caused the development of the tumor.

*Examination of the Tumor.*—There was a small tumor lying in the muscles of the left flank. When the tumor was carefully microscopically examined, it was found to be composed principally of granular debris and a few fibres of finely fibrillative conntive tissue substance. It did not present any of the characters of a sarcoma or carcinoma, but more like the degenerating contents of a hæmatoma.

NEW YORK CITY, August, 1882.

